# Second-Look Needle Arthroscopy After Prior Surgical Treatment for Cartilage Lesions of the Ankle: The Amsterdam and New York City Perspectives

**DOI:** 10.1177/19476035241306550

**Published:** 2024-12-16

**Authors:** Alex B. Walinga, James Butler, Jari Dahmen, Sjoerd A.S. Stufkens, Guillaume Robert, John G. Kennedy, Gino M.M.J. Kerkhoffs

**Affiliations:** 1 Department of Orthopedic Surgery and Sports Medicine, Amsterdam UMC, Location University of Amsterdam, Amsterdam, The Netherlands; 2 Amsterdam Movement Sciences, Sports, Musculoskeletal Health, Amsterdam, The Netherlands; 3 Academic Center for Evidence-Based Sports Medicine (ACES), Amsterdam, The Netherlands; 4 Amsterdam Collaboration on Health & Safety in Sports, International Olympic Committee (IOC) Research Center Amsterdam UMC, Amsterdam, The Netherlands; 5 Department of Orthopedic Surgery, NYU Langone Health, New York, NY, USA

**Keywords:** osteochondral lesions of the talus, second-look needle arthroscopy, NanoScope, autologous osteochondral transplantation, talar osteoperiostic grafting from the iliac crest

## Abstract

**Objective:**

The purpose of this prospective study was to evaluate the quality of the reparative cartilage during second-look needle arthroscopy following talar osteoperiostic grafting from the iliac crest (TOPIC) or autologous osteochondral transplantation (AOT) procedure for the management of large osteochondral lesions (OCLs) of the talus.

**Design:**

Prospective case series.

**Methods:**

Patients who underwent second-look needle arthroscopy following either TOPIC or AOT procedure were prospectively recruited when they needed a second look. The primary outcome was the assessment of the quality of the reparative cartilage via second-look needle arthroscopy scored by the International Cartilage Repair Society (ICRS) score. The secondary outcomes were the number and nature of needle arthroscopy interventions and complications associated with these interventions.

**Results:**

Five patients underwent second-look needle arthroscopy following TOPIC procedure and 11 patients underwent second-look in-office needle arthroscopy following AOT. The mean ICRS in the TOPIC cohort was 9.4 ± 1.0 at a mean time of 24.4 months following the index procedure. The mean ICRS in the AOT cohort was 10.6 ± 1.3 at a mean time of 58.8 months following the index procedure. No complications were observed in either cohort.

**Conclusion:**

This study demonstrated that TOPIC and AOT lead to adequate-looking quality reparative cartilage at short-term to mid-term follow-ups. However, further studies with larger patient cohorts and longer follow-ups are warranted. Furthermore, second-look needle arthroscopy is a safe and viable minimally invasive procedure that can effectively evaluate the quality of reparative cartilage following surgical intervention for OCLs of the talus.

## Introduction

An osteochondral lesion (OCL) of the talus involves injury to the articular cartilage and/or underlying subchondral bone of the talar dome. This pathology is typically preceded by a traumatic event, most commonly by ankle fractures and/or recurrent ankle sprains.^1,2^ The management of OCLs of the talus is primarily dictated by lesion size. Small lesions (<10 mm in diameter or <100 mm^2^ in area) are treated with reparative techniques including arthroscopic debridement, and bone marrow stimulation with or without biological adjuncts. Large lesions (>10 mm in diameter or >100 mm^2^ in area) warrant more extensive surgical intervention in the form of replacement procedures, including talar osteoperiostic grafting from the iliac crest (TOPIC) and autologous osteochondral transplantation (AOT).^3^ TOPIC involves replacement of the OCL with an autograft harvested from the ipsilateral iliac crest. The presence of chondrocyte precursor cells in the periosteum’s cambium layer renders the iliac crest a favorable graft due to its potential for cartilage-like tissue regeneration.^4^ TOPIC has proven to be a reliable, safe technique, producing satisfactory functional results and early radiographic consolidation of the graft on the computed tomography (CT) at short-term follow-up.^4-6^ AOT involves resection of diseased cartilage from the talus which is directly replaced with healthy cartilage from the nonweightbearing portion of the ipsilateral lateral femoral condyle.^7^ This procedure has produced reproducible clinical outcomes and excellent rates of return to sport at mid-term follow-ups.^7-11^

Persistence of anterior ankle pain following surgical intervention for OCLs of the talus can be attributed to poor integration of the reparative tissue, degeneration of the graft, and/or anterior ankle impingement from excessive scar tissue formation or the development of osteophytes.^3^ The re-emergence of needle arthroscopy in recent years offers a promising modality that can visualize and assess the reparative cartilaginous tissue and treat any cause of anterior ankle impingement.^12-14^ Needle arthroscopy involves the use of a 0-degree arthroscope, with a “chip-on-tip” technology to provide a 400 × 400 resolution. The use of a 1.9-mm arthroscope leads to minimal disruption of the soft tissue envelope thereby reducing wound complications and facilitating rapid return to daily activities.^13^ In-office needle arthroscopy (IONA) can be performed under local anesthesia and circumvents the drawbacks associated with arthroscopy performed in a formal operating suite.^15^ The indications for IONA are not only diagnostic capabilities but also therapeutic indications including soft tissue resection, bony exostosis resection, delivery of orthobiologics, and lavage of joint arthritis.^15-19^

Second-look arthroscopic examination following surgical intervention for OCLs of the talus has been previously described.^20,21^ However, the use of needle arthroscopy for second-look arthroscopic examination has not been studied to date. Consequently, the purpose of this study is to evaluate the quality of the reparative cartilage following TOPIC and AOT procedures for OCLs of the talus through second-look needle arthroscopy. In addition, the second purpose of this study is to assess the number and nature of needle arthroscopy-assisted interventions and their safety practicality.

## Methods

This prospective international multicenter observational pilot study was carried out in Amsterdam UMC’s Department of Orthopedic Surgery and NYU Langone Health’s Department of Orthopedic Surgery. The study was conducted in agreement with the 1964 Helsinki Declaration and its later amendments. Approval for the study was granted by the Medical Ethics Committee of the University of Amsterdam, with reference number MEC 08/326. In addition, institutional review board approval was obtained from NYU Langone Health under reference number i23-00716.

### Patient Selection

**Figure 1.**
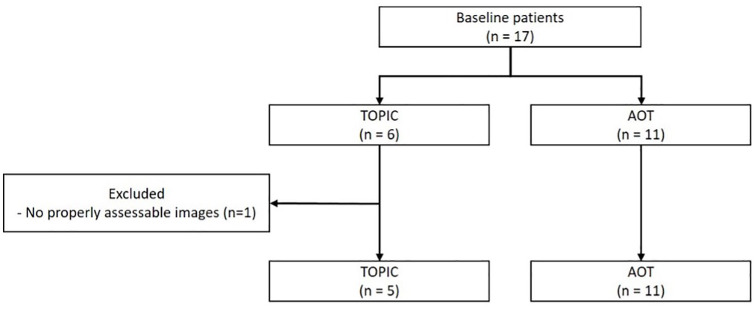
Flowchart of patient selection with inclusion and exclusion criteria.

Patients with ankle complaints following a TOPIC (done in Amsterdam) or AOT (done in NYU) procedure, who were scheduled for a second look, were invited to participate in this study. Patients who underwent second-look needle arthroscopy following either TOPIC or AOT procedure for OCLs of the talus were prospectively included (**Fig. 1**). Patients were excluded if there were no adequately assessable images. In addition, the following variables were extracted: age, gender, body mass index (BMI), laterality, prior surgical intervention, morphology and location of OCL, indication of the second-look needle arthroscopy, duration of second-look needle arthroscopy, and days between prior surgical treatment and second look.

Eleven patients underwent second-look IONA after the AOT procedure. Eleven patients (three females, eight males), with a mean age of 40.5 ± 12.8 years, and a mean BMI of 25.2 ± 2.6 kg/m^2^ were prospectively included in the study. The mean lesion size prior to AOT was 120.9 ± 16.7 mm^2^. The mean lesion volume was 0.6 ± 0.2 cm^3^. There were seven cystic lesions (i.e., osteochondral lesions with subchondral cysts) (63.6%) and one patient (9.1%) had a prior microfracture procedure. The median time between AOT and the second-look IONA was 25.0 months (range, 3.5 to 156.4 months). The indications for second-look IONA included anterior ankle impingement in all (100%) patients (**Table 1**).

**Table 1.** Patient Characteristics.

AOT	Patient	Age	Gender (M/F)	BMI (kg/m2)	Side	Comorbidity	OLT Location	OLT Surface size (mm2)	OLT Volume (cm3)	OLT Morphology	Indication SL	Operation
	1	57	M	24.9	R	Yes	CM and CL	112	0.448	Cystic	Impingement	Debridement and exostoses
	2	43	M	27.6	L	No	PL	132	0.7128	Cystic	Impingement	Debridement and exostoses
	3	40	M	23.7	R	Yes	CM	127	0.7747	Crater	Impingement	Debridement and exostoses
	4	37	F	25.2	R	No	PM	121	0.5445	Cystic	Impingement	Debridement and exostoses and removal of loose body
	5	44	M	26.6	L	No	CM	94	0.3666	Crater	Impingement	Debridement and exostoses and PRP
	6	18	F	20.0	L	Yes	CM and PM	142	0.8804	Cystic	Impingement	Debridement and exostoses
	7	48	M	26.4	L	Yes	CM	107	0.5029	Crater	Impingement	Debridement and exostoses
	8	61	M	26.1	L	No	CM	124	0.5208	Cystic	Impingement	Debridement and exostoses
	9	49	F	29.7	R	Yes	CM	151	0.8909	Cystic	Impingement	Debridement and exostoses and PRP
	10	26	M	25.8	R	No	AL	114	0.627	Crater	Impingement	Debridement and exostoses
	11	32	M	21.1	L	Yes	CL	105	0.609	Cystic	Impingement	Debridement and exostoses
**TOPIC**	1	21	F	33.5	R	No	PM	189	1.06	Cystic	Impingement	Debridement
	2	21	F	19.3	L	No	PM	124	0.67	Crater	ROIF	Inspection and removal of the hardware
	3	71	M	23.6	L	No	CM	200	3.95	Hemicap	Pain	HLA under visualization
	4	22	F	21.2	R	No	CM	133	0.8	Crater	Loose body	Debridement and removal of loose body
	5	35	F	23.1	L	No	PM	200	1.57	Crater	ROIF	Inspection and removal of the hardware

AL = anterolateral; AOT = autologous osteochondral transplantation; BMI = Body Mass Index; CM = centromedial; CL = centrolateral; F = female; HLA = hyaluronic acid; L = left; M = male; N/A = not applicable; OLT = osteochondral lesion of the talus; PM = posteromedial; PL = posterolateral; PRP = platelet-rich plasma; R = right; ROIF = removal of internal; SL = second-look; TOPIC = talar osteoperiostic grafting from the iliac crest.

Six patients underwent second-look needle arthroscopy following TOPIC procedure, of which 1 patient was excluded as there were no adequately assessable intra-articular images/videos. Five patients (four females, one male), with a mean age of 34 years (range, 21 to 71 years), and a mean BMI of 24.1 kg/m^2^ (range, 19.3 to 33.5 kg/m^2^) were prospectively included in the study. The mean lesion size prior to TOPIC procedure was 169.5 ± 33.6 mm^2^. One ankle (20%) had a cystic lesion, three ankles (60%) had a crater lesion, and one ankle (20%) had a Hemicap in the talus before the TOPIC procedure. The median time between the TOPIC and the second look was 27.9 months (range, 8.2 to 41.1 months). The indications for second-look needle arthroscopy included anterior ankle impingement in three patients and removal of symptomatic hardware in two patients (**Table 1**).

### Outcome Measures

#### Primary outcome measure

The primary outcome was the assessment of the quality of the reparative cartilage via second-look needle arthroscopy scored by the International Cartilage Repair Society (ICRS) score for both treatment (TOPIC and AOT) groups^22^ (**Table 2**).

**Table 2.** ICRS Macroscopic Evaluation of Cartilage Repair.

Cartilage Repair Assessment ICRS	Points
Degree of defect repair	
In level with surrounding cartilage	4
75% repair of defect depth	3
50% repair of defect depth	2
25% repair of defect depth	1
0% repair of defect depth	0
Integration to border zone	
Complete integration with surrounding cartilage	4
Demarcating border <1 mm	3
3/4th of graft integrated, 1/4th with notable border >1 mm width	2
1/2 of graft integrated with surround cartilage, 1/2 with a notable border > 1 mm	1
From no contact to 1/4th of graft integrated with surrounding cartilage	0
Macroscopic appearance	
Intact smooth surface	4
Fibrillated surface	3
Small, scattered fissures or cracks	2
Several, small or few but large fissures	1
Total degeneration of grafted area	0
Overall repair assessment	
Grade I: normal	12
Grade II: nearly normal	11–8
Grade III abnormal	7–4
Grade IV: severely abnormal	3–1

#### Secondary outcome measures

The secondary outcomes were the number and nature of needle arthroscopy interventions and complications associated with these interventions.

### Surgical Technique

Needle arthroscopy of the ankle was performed in all patients as previously described.^23^ In the TOPIC cohort, this procedure was performed in a formal operating suite under general anesthesia with the use of a pneumatic tourniquet applied to the proximal thigh. In the AOT cohort, this procedure was performed in the office setting (IONA) with a local intra-articular injection of lidocaine, bupivacaine, and epinephrine without the use of a pneumatic tourniquet.

All patients were positioned supine. The relevant surface anatomy, the course of the superficial peroneal nerve, and standard anteromedial and anterolateral portals were marked out. Saline solution was used to distend the joint, and a distraction soft tissue strap was applied when necessary.

The arthroscopy portals were made using a number 11-blade.^23^ A small 2-mm stab incision is made to accommodate the 1.9-mm needle-arthroscope with zero-degree inclination (NanoScope; Arthrex, Naples, FL). A blunt trocar is then used to access the joint. The camera is exchanged over the trocar and connected to an integrated fluid management system at a pressure of 35 mm Hg or to fluid syringes. A diagnostic arthroscopy is performed, alternating between anteromedial and anterolateral portals, allowing visualization of various anatomical structures. In cases of identified anterior ankle impingement, a 2.0-mm or 3.0-mm shaver is employed to optimize visualization by removing scar tissue and synovial hyperplasia. Any identified osteophytes were resected using the 2.0-mm or 3-mm burr if necessary. Portals are closed using adhesive wound closure strips, and a dry sterile dressing is applied to facilitate early ankle motion.

### Postoperative Protocol

In the TOPIC cohort, patients remained partially weightbearing on crutches for 3 to 5 days followed by gradual increase in weightbearing as tolerated. In the AOT cohort, patients were allowed to be fully weightbearing as tolerated immediately using a rigid postoperative shoe. Ankle pumps and circumduction exercises were prescribed every hour for 5 min during the initial 24 h.

### Power Calculation

The study aimed to maximize its inclusion of patients by enrolling the highest number possible from the prospective cohort. In this regard, it’s important to note that no predefined sample size calculation was conducted prior to the commencement of the study. Instead of using a predetermined sample size, the study embraced an inclusive approach, allowing for the incorporation of as many eligible patients as feasible within the available prospective cohort.

### Statistical Analysis

The primary and secondary outcome measures were analyzed separately for both treatment groups, as the indications for these treatments differ. The primary outcome measure was independently and blindly assessed by two research fellows using the ICRS score, meaning the researchers did not know which images corresponded to which patient. Discordant judgment in determining the ICRS was resolved through discussion by the senior orthopedic surgeon and two research fellows until consensus was made. The secondary outcome measures were summarized using descriptive statistics. Categorical variables were represented using absolute numbers and percentages. For continuous variables, means with standard deviations were reported if they exhibited a normal distribution. In cases where the distribution was nonnormal, median values with interquartile ranges (IQRs) were used for expression.

## Results

### AOT

#### Primary outcome: the appearance of cartilage repair

**Figure 2.**
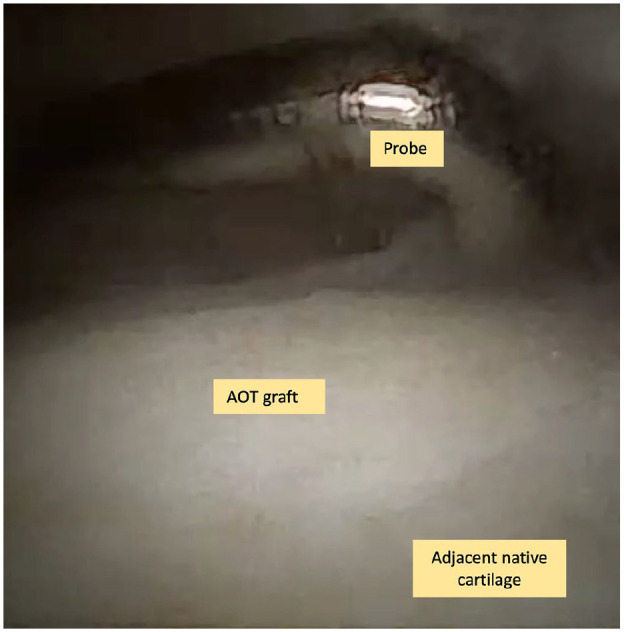
Incorporated AOT graft seen from the needle arthroscopy.

The mean ICRS score across the 11 patients was 10.6 ± 1.3 (**Table 3**). Three patients (27.3%) were graded as normal (grade 0, **Fig. 2**) and eight patients (72.7%) were graded as nearly normal (grade I). With regard to “degree of defect repair,” eight grafts (72.7%) demonstrated repaired tissue at the level of the surrounding cartilage and three grafts (27.3%) demonstrated 75% repair of defect depth. With regard to “integration to the border zone,” eight grafts (72.7%) demonstrated complete integration with adjacent cartilage, three grafts (27.3%) demonstrated a demarcating border of less than 1 mm. With regard to “macroscopic appearance,” three grafts (27.3%) had an intact smooth surface, seven grafts (63.6%) demonstrated some fibrillated surfaces, and one graft (9.1%) demonstrated small macroscopic cracks or fissures (**Table 3**).

**Table 3.** ICRS Assessed During Second-Look Needle Arthroscopy AOT.

Patient	Degree of Defect Repair	Integration to Border Zone	Macroscopic Appearance	Overall Repair Assessment
1	4	4	3	11
2	4	4	3	11
3	3	4	3	10
4	4	3	3	10
5	3	4	3	10
6	4	4	4	12
7	3	3	2	8
8	4	4	4	12
9	3	3	3	9
10	4	4	4	12
11	4	4	3	11

AOT = autologous osteochondral transplantation; ICRS = International Cartilage Repair Society.

#### Secondary outcome: the nature and number of needle arthroscopy interventions and their indications

A needle arthroscopic intervention was performed in 11 ankles (100%). In 11 ankles, impinging soft tissue structures and loose bodies were debrided and resected. In addition, in two ankles platelet-rich plasma (PRP) was injected under visualization of the needle arthroscope. The mean operation time of the second-look was 37.7 ± 4.9 min. No major or minor complications were identified related to the second-look IONA.

### TOPIC

#### Primary outcome: the appearance of cartilage repair

**Figure 3.**
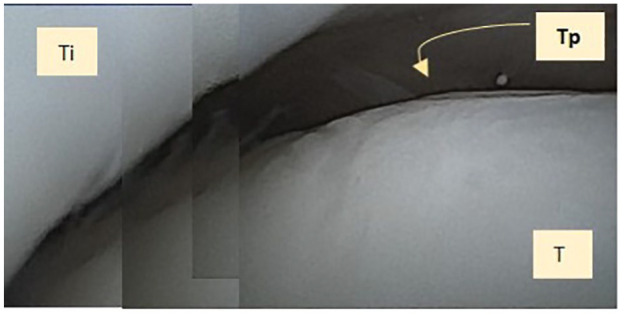
Incorporated TOPIC graft composite image seen from the needle arthroscopy. T = talus; Ti = distal tibia; Tp = TOPIC graft.

The mean ICRS score across the five patients was 9.4 ± 1.0 (**Table 4**). The overall repair assessment of all ankles was graded as nearly normal (grade I, **Fig. 3**) in 100% of patients. With regard to “degree of defect repair,” 100% of the lesions demonstrated repaired tissue at the level of the surrounding cartilage. With regard to “integration to the border zone,” one graft (20%) demonstrated complete integration with adjacent cartilage, three grafts (60%) demonstrated a demarcating border of less than 1 mm, and one graft (20%) demonstrated 3/4th of graft integrated, with 1/4th of a notable border of more than 1 mm width. With regard to “macroscopic appearance,” no grafts had an intact smooth surface, two grafts (20%) demonstrated some fibrillated surfaces, and three grafts (60%) demonstrated small macroscopic cracks or fissures (**Table 4**).

**Table 4.** ICRS Assessed During Second-Look Needle Arthroscopy TOPIC.

Patient	Degree of Defect Repair	Integration to Border Zone	Macroscopic Appearance	Overall Repair Assessment
1	4	3	2	9
2	4	4	3	11
3	4	3	3	10
4	4	2	2	8
5	4	3	2	9

ICRS = International Cartilage Repair Society; TOPIC = talar osteoperiostic grafting from the iliac crest.

#### Secondary outcome: the nature and number of needle arthroscopy interventions and their indications

A needle arthroscopic intervention was performed in three ankles (60%). In one ankle, hyaluronic acid was injected under visualization of the needle arthroscope, and in two ankles (40%) impinging soft tissue structures and loose bodies were debrided and resected. The mean operation time of the second-look was 51.4 ± 19.7 min. No major or minor complications were identified related to the second-look needle arthroscopy.

## Discussion

The most important findings of this study were that AOT and TOPIC lead to high-quality reparative cartilage at short-term to mid-term follow-up. This study demonstrated that second-look needle arthroscopy is a safe and feasible minimally invasive procedure that can effectively evaluate the quality of reparative cartilage following surgical intervention for OCLs of the talus. In addition, the utility of IONA extends past its diagnostic capabilities and can be utilized to concomitantly treat both bony and soft tissue sources of anterior ankle impingement in the office setting.

Second-look arthroscopic evaluation following surgical intervention for OCLs of the talus has been well described in the literature.^20,21^ These procedures are typically performed with a standard 4.0-mm arthroscope in a formal operating suite. However, numerous drawbacks exist for this procedure including excessive trauma to the soft tissue envelope, and additional use of a distractor to gain access to the joint.^3^ Needle arthroscopy is an innovative diagnostic and treatment strategy that mitigates the challenges and limitations associated with traditional ankle arthroscopy. The 1.9-mm arthroscope limits the trauma to the surrounding soft tissue structures and facilitates rapid return to sporting and daily activities. In addition, this procedure can be conducted in the office setting with wide awake local anesthetic with no tourniquet which mitigates against risks associated with general anesthesia and sedation and extensive operation and facility fees for the patient.^15,23^ This study describes the first utilization of second-look needle arthroscopy following surgical intervention for OCLs of the talus. Needle arthroscopy accurately quantified the degree of cartilage repair following TOPIC and AOT procedures and permitted resection of impinging bony and soft tissue structures. No complications were observed in both patient cohorts illustrating that second-look needle arthroscopy is a safe, reliable, and effective tool in the evaluation of the quality of the reparative cartilage following TOPIC or AOT procedures.

OCLs of the talus are a difficult-to-treat pathology primarily due to the poor regenerative capacity of the articular cartilage.^24^ The use of autologous osteoperiosteal grafts from the iliac crest for large OCLs of the talus was initially described by Hu *et al*.^25^ who reported satisfactory clinical outcomes at short-term follow-up. However, the cylindrical plugs utilized in these studies are limited due to their fixed size which may not allow for a perfect, press fit at the recipient talus. The TOPIC procedure was first described by Kerkhoffs *et al*.^4^ in 2021 to circumvent the limitations with the use of cylindrical plugs. For lesions located at the medial talar dome, a medial distal tibial osteotomy is performed to gain access to the lesion. The lesion is excised with an oscillating saw and the base of the defect is microdrilled using a 2.0 mm drill. The size of the defect is measured and the autograft from the iliac crest is harvested with an oscillating saw. The shape of the graft is adjusted so that it lies perfectly flush with the adjacent native cartilage. Dahmen *et al*.^6^ reported on the outcomes of this procedure at 2-year follow-up in a prospective cohort of 44 patients with a mean lesion size of 177 mm^2^. The authors reported excellent subjective clinical outcomes in their cohort together with consolidation of the graft in 100% of patients on postoperative CT imaging.

Large lesions are most adequately treated with procedures that directly replace the osteochondral unit, such as AOT.^7^ AOT was described by Kennedy and Murawski^7^ in a retrospective case series of 72 patients who underwent AOT at 28 months follow-up. Excellent functional outcomes exhibited by postoperative FAOS scores of 28 months and a mean return to sport time of 12 weeks were reported. Furthermore, Flynn *et al*.^26^ performed postoperative MRIs on a cohort of 61 patients who underwent AOT at 24.8 months follow-up to evaluate both the quantitative and qualitative characteristics of the graft tissue. Qualitative assessment of the repair tissue was assessed via magnetic resonance observation of cartilage repair tissue (MOCART) scores yielding a mean score of 85.8. Quantitative assessment was conducted via T2-mapping, which demonstrated comparable deep graft relaxation values in the repair tissue (30.9 s) and the adjacent native cartilage (30.0 s; *P* = 0.305). The results of this prospective study demonstrated excellent ICRS scores (10.6 ± 1.3) at a mean 58.8-month follow-up in the AOT cohort, suggesting that the quality of the cartilage lining the graft is maintained at mid-term follow-up. Our findings are consistent with the current literature regarding second-look arthroscopy following AOT. Harada *et al*.^20^ conducted a retrospective study of 10 patients (12 ankles) who underwent second-look anterior ankle arthroscopy at a mean time of 12.1 months following AOT. The mean ICRS score in their cohort was 11.4 with 8 cases of grade I, 4 cases of grade II, and no cases of grade III or grade IV. In addition, their mean American Orthopedic Foot & Ankle Society (AOFAS) score at final follow-up was 98.1 ± 2.8 reflecting excellent subjective clinical outcomes at the time of second-look arthroscopy.

This is the first study evaluating second-look arthroscopic examination following TOPIC procedure for OCLs of the talus. The mean ICRS in this cohort was 9.4 ± 1.0 with all ankles graded as grade I, indicating satisfactory reparative tissue. It is concerning that only 1 lesion demonstrated complete integration with the adjacent native articular cartilage, potentially indicating that the longevity of the TOPIC procedure may be compromised. However, this finding must be interpreted in light of the small patient cohort in this study. Given the small sample size in both the AOT cohort and TOPIC cohort, it is outside the scope of this study to determine the superiority in cartilage repair between either technique. Thus, further studies with larger patient cohorts collected patient-reported outcomes measurements (PROMs), and postoperative MRIs with T2-mapping are warranted.

In total, 87.5% of patients were indicated for second-look needle arthroscopy due to anterior ankle impingement, which is a common finding after invasive ankle procedures. In particular, concentrated bone marrow aspirate which was utilized in all patients who underwent AOT has been shown to have a propensity for the development of excessive scar tissue due to the presence of the TGF-B1 isoform.^27^ Thus, IONA is an attractive treatment strategy in this setting as it can effectively resect the impinging scar tissue and osteophytes with minimal downtime in recovery. A recent retrospective study by Colasanti *et al*.^28^ recorded outcomes following the use of IONA for anterior ankle impingement utility of IONA in the debridement. A total of 31 patients were included in this study which found significant improvement in Patient-Reported Outcomes Measurement Information System (PROMIS) scores and FAOS scores at 1-year follow-up, with 96% of patients returning to sport at a mean time of 3.9 weeks.

The findings of this study must be considered in the context of its limitations. First, the relatively small number of patients included may not adequately reflect the true outcomes of cartilage repair quality observed with needle arthroscopy following TOPIC or AOT treatment. This limitation restricts our ability to generalize these results to a broader patient population. Second, it is important to acknowledge the possibility of selection bias within our patient cohort, which could introduce a bias toward those who opted for this particular intervention. To comprehensively assess the utility of needle arthroscopy for various second-look scenarios, larger studies with extended follow-up periods will be required. There was diversity in the needle arthroscopy settings, as IONA was conducted within the AOT cohort, while needle arthroscopy took place in a dedicated operating suite within the TOPIC cohort. Third, one patient treated with AOT had a second-look procedure after 3.5 months, which might be too early for an accurate assessment of the cartilage. However, this applies to only one individual, as the median follow-up time is 25 months. For the majority of the group, the time since surgery is much longer than 3.5 months. Fourth, in some cases, obtaining a complete view of the integrated cartilage repair, particularly in the posterior aspect of the defect, can be challenging. However, second-look arthroscopy is still considered by some to be the gold standard for assessing the quality of cartilage lesions in the ankle [ref]. As a minimally invasive procedure, it remains a safe and viable method for evaluating the quality of the repair.

## Conclusion

This study demonstrated that AOT and TOPIC lead to adequate-quality reparative cartilage at short-term to mid-term follow-up, but further studies with larger patient cohorts and longer follow-ups are warranted. In addition, second-look needle arthroscopy is a safe and viable minimally invasive procedure for assessing the quality of reparative cartilage subsequent surgical intervention for OCLs of the talus. In addition to arthroscopic evaluation of the reparative cartilage, this procedure has therapeutic capabilities and can resect bony and soft tissue sources of anterior ankle impingement.
